# Informatics for COVID-19 in New York and California

**DOI:** 10.1017/dmp.2021.53

**Published:** 2021-02-16

**Authors:** Seungil Yum

**Affiliations:** Design, Construction, and Planning, University of Florida, Gainesville, FL

**Keywords:** California, coronavirus, COVID-19, New York, social network analysis

## Abstract

**Objective:**

This study explores how social networks for coronavirus disease (COVID-19) are differentiated by regions.

**Methods:**

This study employs a social network analysis for Twitter in New York and California.

**Results:**

National key players play an important role in New York, whereas regional key players exert a significant impact on California. Some key players, such as the US President, play an essential role in both New York and California. Hispanic key players play a crucial role in California. Each group is more likely to show communication networks within groups in New York, whereas it is more apt to exhibit communication networks across groups in California. Government players play a different role in social networks according to regions.

**Conclusions:**

Governments should understand how social networks for COVID-19 are differentiated by regions to control the ongoing pandemic effectively.

## Introduction

Coronavirus disease (COVID-19) has become a serious disease in human history. According to the Johns Hopkins Coronavirus Resource Center, as of December 4, 2020, 65 359 887 cases of COVID-19 have been reported in more than 188 countries, resulting in 1 509 141 deaths. The United States experiences the most serious cases of the COVID-19 pandemic. As of the same date, there are 14 148 719 cases and 276 401 deaths in the United States, representing nearly one-fifth of the world’s COVID-19 cases and deaths, and the most cases and deaths across the world.^1^

Scholars have highlighted the characteristics and effects of COVID-19 by employing a social network analysis (SNA). For example, Yum^2^ highlights that President Trump plays the most significant role in social networks of COVID-19 for both in-degree centrality and content in 2864 Twitter users and 2775 communications of Twitter. Taylor et al.^3^ show worry, avoidance, and coping issues during the COVID-19 pandemic, based on a sample of 3075 American and Canadian adults who completed an online survey.

However, prior studies have barely explored how people show different social networks for COVID-19 across regions. People would build specific social networks for COVID-19, according to regions, since they have respective regional characteristics. Thus, this study aims to highlight how social networks for COVID-19 are differentiated by regions.

To the best of my knowledge, no articles have highlighted how people build online social networks for COVID-19 according to different US regions. Therefore, this study highlights how people communicate with each other and share relevant information on COVID-19 in New York and California. These are 2 of the top states of confirmed COVID-19 patents in the United States.

## Research Methodology

This study employs SNA to examine how social networks for COVID-19 are differentiated by New York and California. SNA explores the behavior of people at the micro level, the pattern of relationships at the macro level, and the interactions between these 2.^4^ This study uses Twitter data to demonstrate social networks of people for COVID-19 across key players, such as institutes, politicians, and organizations. This study observes Twitter data stream between June 11 and June 18, 2020, based on the keywords *COVID-19* and the states, and chooses the best data set for the analyses (June 17 and June 18), based on some important criteria (eg, the number of Twitter users, communications, and suitable content). The descriptive statistics are (1) New York: 17 861 (vertices) and 21 707 (unique edges), and (2) California: 17 191 (vertices) and 19 808 (unique edges).

This study employs NodeXL to highlight the social networks of New York and California for COVID-19. NodeXL is a visualization software program that supports social networks and content analysis. Scholars employ NodeXL to collect, visualize, and analyze social networks based on the elements of graph structures, such as edges and nodes. NodeXL provides various visualization tools and graph drawing layout algorithms, such as Fruchterman-Reingold, Harel-Koren, horizontal sine wave, and vertical sine wave. NodeXL has been extensively used for collecting data from a variety of social media platforms, such as Twitter, Facebook, YouTube, Flickr, and MediaWiki.

This study employs the betweenness centrality to find the top 20 key players for Twitter users. Betweenness centrality captures the number of shortest paths that pass through the target node.^5^ The higher the top key players’ betweenness centrality, the higher of importance they play a hub role in social networks for COVID-19. The betweenness centrality is calculated as follows:
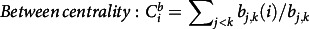



Where C_i_ is the centrality of node i, bj_,k_ = the number of the shortest links that pass through the node between j and k, and b_j,k_ (i) = the number of the shortest links that pass through the node between j and k, going through i.

Next, this study employs a cluster analysis by using the Clauset–Newman–Moore cluster algorithm. Cluster analysis is a methodology for the task of assigning a set of objects into groups so that the objects in the same cluster are more similar to each other than those in other clusters. Cluster analysis allows authors to explore data sets and identify them into relatively homogeneous clusters so that the between-group variation is maximized and the within-group variation is minimized. The clusters should have individuals that show common characteristics in the group and are as different as possible from those in other groups.

The Clauset-Newman-Moore cluster algorithm calculates a sparse matrix to store the current cluster labels and a max-heap of nodes that are un-clustered and their modularity improvement score.^6^ This is because other traditional algorithms, such as the Kernighan–Lin algorithm, spectral partitioning, or hierarchical clustering, work well for some specific cases but perform poorly in more general types. In contrast, the Clauset-Newman-Moore cluster algorithm is remarkably faster than most well-known other algorithms and allows people to visualize huge social networks.^7^ The equation is as follows:
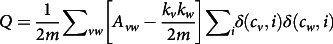



Where Q is the modularity, 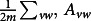 is the number of edges in the graph, v and w are nodes, K_v_ is the degree of a node v, δ function δ(i, j) is 1 if i = j and 0, otherwise, and C is the community.

## Results

**Figure 1.**
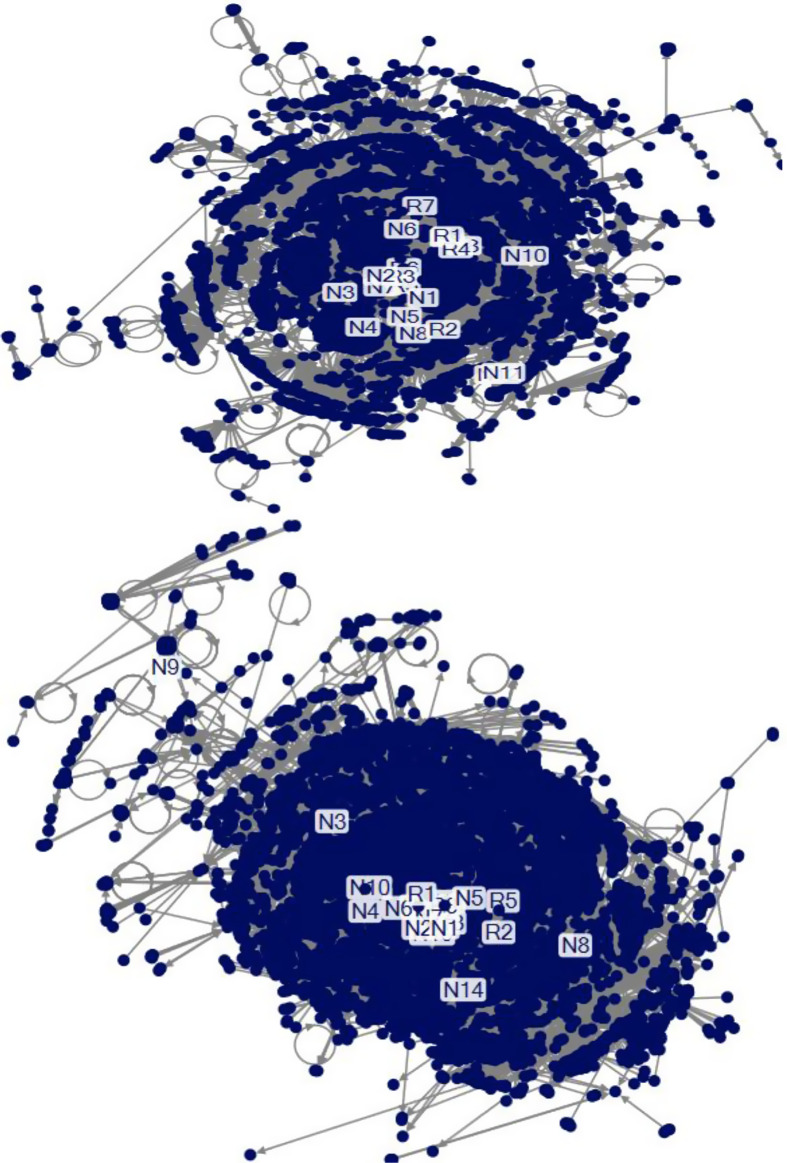
Betweenness centrality (top: New York, bottom: California).

Figure 1 highlights that the betweenness centrality of COVID-19 is differentiated by regions. Social networks in New York show a more dispersed pattern, whereas those in California reveal a relatively dense pattern. Table 1 shows the top 20 public key players of New York and California. They show different characteristics of social networks for COVID-19. National key players play a more important role in New York, whereas regional key players exert a more significant impact on California. For instance, national key players rank from first to fourth in the New York networks, whereas regional key players play the first and fourth significant role in the California networks. Some key players play an important role in both New York and California. For instance, Donald Trump who is the US President ranks eighth in New York and 10th in California.

**Table 1.** The top 20 key players

	New York			California		
	L	BC	Name	L	BC	Name
1	N1	59780670	ABC News	R1	53190482	Los Angeles Times
2	N2	49241290	Bloomberg Opinion	N1	39541828	NBC News
3	N3	29707016	NowThis	N2	33282381	CBS News
4	N4	23062492	Good Morning America	R2	26667199	Gavin Newsom
5	R1	14508074	Andrew Cuomo	N3	15112609	REFORMA Nacional
6	N5	9785664	ABC News Politics	N4	12943634	Bloomberg Opinion
7	R2	8750945	New York Post Metro	N5	10930894	Foro_TV
8	N6	7027420	Donald Trump	N6	8689895	MarketWatch
9	R3	4296038	New York Daily News	N7	6647637	CNN
10	N7	4195105	The Daily Beast	N8	5800422	Donald Trump
11	N8	3468089	This Week	N9	5471606	EFE Noticias
12	R4	2942606	City of New York	N10	5299887	ABC7 Eyewitness News
13	N9	2695858	YouTube	R3	4958622	CA Public Health
14	N10	2613572	Daily Mail Online	N11	3371476	The COVID Tracking Project
15	R5	2406519	NBC New York	N12	3074452	New Day
16	R6	2399926	New York Post	R6	2399926	New York Post
17	N11	2352404	Franklin Graham	N11	2352404	Franklin Graham
18	N12	2205518	Samaritan’s Purse	N12	2205518	Samaritan’s Purse
19	R7	1832049	The New York Times	R7	1832049	The New York Times
20	R8	1771860	Bill de Blasio	R8	1771860	Bill de Blasio

*Notes*: L = label; BC = betweenness centrality; R = regional key players; N = national key players.

Next, the channels of ABC News play a crucial role in the New York networks. In contrast, Hispanic key players play an important role in California. This is because California has the largest population of Hispanic people in the United States. According to the World Population Review, as of 2020, California has a Hispanic population of 15 477 000, which is about 4 times higher than that of New York (3 811 000).^8^

**Figure 2.**
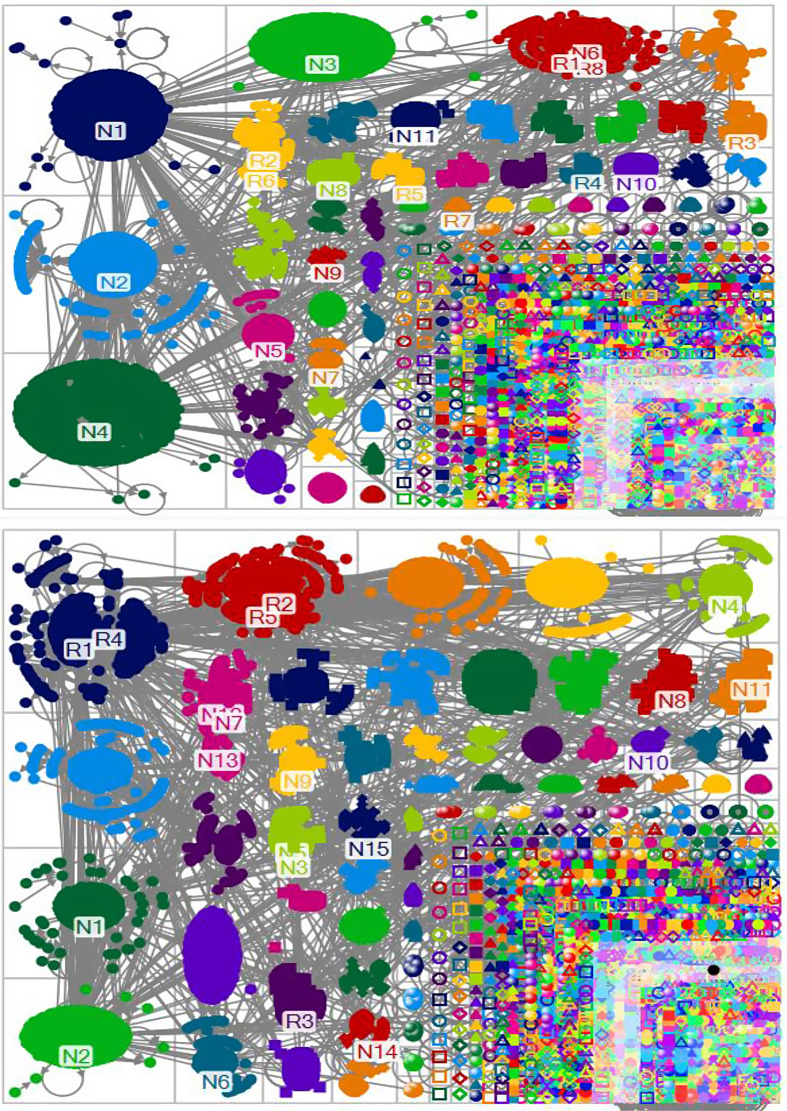
Social networks according to groups (top: New York, bottom: California).

Figure 2 shows that a national key player (ABC News) plays an important role in the largest group (group 1) of the New York networks. In contrast, regional key players (Los Angeles Times [R1] and KTLA [R4]) play a central role in the largest group of the California networks. The figure also highlights that each group is more likely to show communication networks within groups in New York, whereas it is more apt to exhibit communication networks across groups in California.

Table 2 shows that government key players play a different role in New York and California. For example, Andrew Cuomo (governor), Bill de Blasio (mayor), and Donald Trump (President) exert a significant impact on the same group (group 5) in the New York networks. In contrast, government players in California are located in different groups according to regional and national key players.

**Table 2.** National and regional key players by groups

G	New York	G	California
1	ABC News	1	Los Angeles Times (R), KTLA (R)
2	Bloomberg Opinion	3	NBC News
3	Good Morning America	4	CBS News
4	NowThis	5	Gavin Newsom (R), Office of the Governor of California (R)
5	Andrew Cuomo (R), Bill de Blasio (R), Donald Trump	8	Bloomberg Opinion
7	New York Post Metro (R), New York Post (R)	9	CNN, New Day, ABC News
9	ABC News Politics	12	MarketWatch
13	Franklin Graham, Samaritan’s Purse	17	Donald Trump
18	New York Daily News (R)	18	The COVID Tracking Project
19	NBC New York (R)	19	EFE Noticias
20	This Week	20	REFORMA Nacional, Foro_TV
23	Daily Mail Online	22	CA Public Health (R)
24	City of New York (R)	25	Rolling Stone
29	YouTube	29	Daily Mail US
30	The Daily Beast	35	ABC7 Eyewitness News
42	The New York Times (R)		

*Notes*: G = group; (R) = regional player.

## Discussion

The impact of COVID-19 is rapidly changing. The numbers of COVID-19 patients and deaths on December 4, 2020, are 5.3 times and 2.8 times higher than those of June 18, 2020. This is because COVID-19 has a high reproduction number, and there have been second wave (or third) COVID-19 pandemics across the world. For instance, the United States has reached a new record high in the number of daily COVID-19 infections on October 23 with 83 757 cases, surpassing the peak in mid-July.^9^

While this study employs SNA on June 17 and June 18, many government policies and responses would impact social networks for COVID-19 after June, given that the social networks are privy to changes. For instance, on June 18, the New York Government announced the government policy that New York City was on track to enter Phase 2 of reopening on June 22. The California Government issued universal masking guidance on June 18 to require the wearing of cloth face coverings by all individuals in all public indoor settings. Therefore, scholars should explore the impacts of COVID-19 on human life at various periods to consider the changes in government policies, social networks, and COVID-19 responses.

The US Government has strengthened the social networks for COVID-19 since June. For example, the Federal Communications Commission ensures that Americans stay connected during the COVID-19 pandemic, the General Services Administration provides tips for making agency communications accessible to everyone, and the US Agency for Global Media covers the coronavirus pandemic for communications with the public.^10^

This study suggests implications for the broader public health and COVID-19 response as follows: governments and public health agencies should analyze key players in their region because they play a different role in social networks according to regions. Some key players, such as the US President, play an important role across regions. Therefore, it would be an effective way to use these players to spread important information on COVID-19. Policy-makers and health planners should understand the characteristics of their regions, such as the racial and ethnic composition, to understand online social networks since key players are highly associated with them. Government key players should analyze their role in social networks for COVID-19 because they are located in different groups. SNA for social network services (SNS) can be employed for public health agencies, policy-makers, and the Centers for Disease Control and Prevention to understand COVID-19 responses among people.

## Conclusions

This study finds that social networks for COVID-19 are significantly differentiated by regions. To be specific, national key players play a more important role in New York, while regional key players exert a more significant impact on California. Some key players, such as Donald Trump, ABC News, and Bloomberg Opinion, play an important role in both New York and California, but they exert a different impact on them. Hispanic key players play an important role in California. Each group is more likely to show communication networks within groups in New York, while it is more apt to exhibit communication networks across groups in California. Government players in New York play an important role in the same group networks, whereas those in California are located in different group networks.

This study has some limitations as follows: this study uses the data between June 17 and June 18, whereas social networks would be different since June, given the rapid pace of COVID-19. This study explores the Twitter data only, thus other SNS, such as Facebook or YouTube, would show different results for social networks. This study selects 2 US states, whereas other states would show different characteristics of social networks for COVID-19. Future research should explore social networks for COVID-19 based on a multitude of periods, SNS, and regions.
